# Lessons to Europe from China for cancer treatment during the COVID-19 pandemic

**DOI:** 10.1038/s41416-020-0856-0

**Published:** 2020-05-14

**Authors:** Ling Peng, Jin-Song Yang, Justin Stebbing

**Affiliations:** 1 Department of Radiotherapy, The First Affiliated Hospital, School of Medicine, Zhejiang University, Hangzhou, Zhejiang Province China; 2 Division of Cancer, Department of Surgery and Cancer, Imperial College London, London, UK

**Keywords:** Cancer, Oncology

## Abstract

During the COVID-19 era, Chinese hospitals have developed a system that enables vulnerable cancer patients to continue to receive high-quality medical care, optimising their survival whilst protecting them. This includes use of digital quick codes, fever clinics and optimal scheduling. We wish to share our experiences working with patients during the pandemic.

## Main

While cancer patients might have a higher risk of harm compared with patients without cancer if infected with SARS–CoV2,^1,2^ the degree of elevated risks if any remains uncertain and will be based on numerous host-, tumour- and treatment-related factors. Compared with the European medical system, there are some measures worth learning from China, from our experience working with cancer patients during the pandemic.

**Fig. 1:**
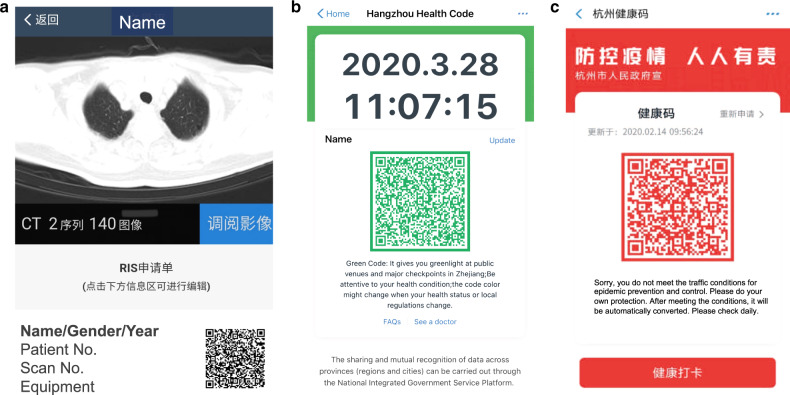
The application of two-dimentional code and health code during the COVID-19 pandemic. **a** Two-dimensional code that Chinese hospitals offered to patients with representative images shared between the patient and the hospital. **b** Health code green indicates that the person is free of contagion risk. **c** Health code red indicates that one came from an area with a higher-than-normal degree of coronavirus infection.

All Chinese hospitals have developed detailed guidelines for protecting potentially vulnerable cancer patients under different conditions and have rearranged non-urgent consultations to minimise a given cancer patient’s exposure to any health care setting; this would be akin to the European experience. In China, hospitals offer two-dimensional barcodes, a so-called QR code (Fig. 1a), for patients to check their lab results and imaging findings simultaneously with medical staff by smartphones, or after it has been viewed by a physician. For those who need to attend to hospital, there are a number of measures to detect possible signals of COVID-19, especially on oncology wards. Wearing masks is required for everyone, whether symptoms exist or not; we are not questioning evidence for this, but psychologically, it serves as a warning to “be careful”. Temperature monitoring is one of the prerequisites in the entrance of every hospital and on every ward and this includes staff. Everyone entering our hospital is mandated to have an Alibaba “health code” that is connected to a large database, providing automatically generated classifications of suspected patients with different “security levels” defined by colour category (Fig. 1b). Only one room in an oncology ward can accommodate a single patient, and visitors for patients were not allowed at any time to enter wards during the “COVID-19 period”. Chinese hospitals have “fever clinics” to effectively screen suspicious COVID-19 patients, and this occurred rapidly for those with cancer with results within 2 h with both PCR and immunoglobulin (IgG and IgM testing). Thus, if a cancer patient with fever attends hospital, they must initially consult an infectious disease specialist; only if COVID-19 is then ruled out, can subsequent diagnostic procedures then be initiated. The hospital I (LP) work at is the designated provincial hospital in Zhejiang Province to admit COVID-19 patients, and the symptoms of patients in Zhejiang Province have on the whole been milder compared with those in Wuhan.^3^ In our department, the number of cancer patients receiving radiotherapy in February 2020 decreased to 35% compared with February 2019; similar figures apply to chemotherapy. We believe overall, however, that both capacity and efficiency of Chinese hospitals have offered cancer patients great support during the COVID-19 era.

For example, one patient we cared for was diagnosed with locally advanced nasopharyngeal cancer, but his “health code” was red (Fig. 1c), indicating that he came from an area with a higher-than-normal degree of coronavirus infection. According to national regulations, medical isolation was required for 14 days after his negative coronavirus test, but the stage of the tumour mandated some urgency. In this case, we first screened him, including the nucleic acid test of swabs and a chest CT scan to rule out the possibility of COVID-19 infection. Then we opened a special room for him with sterilisation procedures and administered induction chemotherapy on the same day. After the chemotherapy was complete, he went to a close-by hotel for self-isolation. At the end of the isolation period of 14 days, he was ready to receive a second cycle of induction chemotherapy.

China’s oncology community has taken measures in response to the virus outbreak. Virtual online hospitals and home drug delivery programmes have helped cancer patients derive access from high-quality medical services. Online medical counselling and timely identification of critical cases are provided by oncologists and medical care staff such as the Patient Education Committee of the Chinese Society of Clinical Oncology (CSCO). Apps, including WeChat groups for cancer patients, are set up, and these offer easy and convenient feedback to doctors. Symptoms such as treatment-related adverse events would receive prompt attention. After cancer patients have returned to their homes following treatment, the community monitors and manages the patients through facial recognition or QR code. If anything untoward occurs, there is active communication with the hospital.

Chinese scholars have developed an optimal scheduling method by a mixed integer programing model using the standard linear solver CPLEX optimiser, which can minimise the waiting time before radiotherapy and decrease chances of cross-infection, thus allocating cancer patients receiving radiotherapy more efficiently (unpublished data). Traditional Chinese Medicine (TCM) is a complementary and alternative part of cancer treatment in China,^4^ and patients prefer to take oral Chinese herbs or intravenous TCM infusions to treat their diseases. TCM has been used routinely and in clinical trials in COVID-19 patients,^5^ such as the botanical extract sophorcarpidine, which was approved as a cancer treatment by the NMPA (National Medical Products Administration), and has preliminary efficacy reported in 40 COVID-19 patients.^6^

Cancer treatment in the era of COVID-19 faces various uncertainties. China has set an example for developing a highly efficient and feasible system that enables the majority of cancer patients to receive high-quality medical care in the COVID-19 era. With such precautions, the cancer community has not been profoundly affected in China.

## Data Availability

Not applicable.
